# Involvement of TGFβ signaling pathway in oxidative stress and diabetic retinopathy

**Published:** 2021

**Authors:** Reanna Rodriguez, Kristine Lowe, Megan Keniry, Andrew Tsin

**Affiliations:** 1 Department of Biology, The University of Texas Rio Grande Valley, Edinburg, TX, United States; 2 School of Medicine, The University of Texas Rio Grande Valley, Edinburg, TX, United States

**Keywords:** Diabetic retinopathy, 661W cone photoreceptors, Vascular endothelial growth factor, Transforming growth factor beta, Hypoxia, Oxidative stress

## Abstract

Diabetic Retinopathy (DR) is a leading cause of blindness in the U.S. However, not much is known of underlying molecular mechanism and how oxidative stress contributes to its development. In the present study, we investigated the involvement of TGFβ signaling pathway on the effect of oxidative stress on VEGF secretion and viability of retinal cells. VEGF is the hallmark that exacerbates DR progression in prolonged diabetes. Some major concerns that have arisen are the underlying effects of antioxidants in elevating VEGF secretion in diabetes. In this study, we evaluated how hypoxia (or low oxygen) impacts viability and VEGF secretion using 661W cone photoreceptor cells. Confluent 661W cells were grown in 5.5 mM normal or 30 mM high glucose, as well as subjected to CoCl_2_ to induce hypoxia. After treatment for 24 hours, conditioned media were collected for ELISA measurement to determine the amount of protein (VEGF) secretion. Viable cell numbers were also recorded. High glucose did not induce significant changes in viable cell number nor VEGF concentration in cell media. However, hypoxia condition resulted in a three-fold decrease in viable cell numbers and a three-fold increase in VEGF concentration. Furthermore, treatment with two TGFβ inhibitors: SMAD 3, SIS (or Inhibitor 1) and TGFβ receptor 1 kinase inhibitor (or Inhibitor 2) resulted in a reversal of hypoxia-induced changes. These results strongly suggest that TGFβ signaling pathway mediates hypoxia-induced retinal cell viability and VEGF secretion. Further translational research studies will provide evidence to identify appropriate and effective pharmaceutical targets in this molecular pathway to mitigate the development of DR.

## Introduction

Diabetes mellitus (DM) is one of the most common chronic-degenerative diseases in the adult populations; type 1 is characterized by the inability to produce the typical amounts of insulin and type 2 diabetes is identified by the body’s reduced sensitivity to insulin [1]. Diabetes is affected by oral intake of high glucose foods, which is found in a variety of commonly consumed foods. Due to prolonged hyperglycemic condition, there is evidence to suggest significant increased levels of various inflammatory responses and in antioxidants are protective of diabetic conditions. Prevalence of diabetes in the world was 6.4% of the population, however, researchers estimate that the prevalence of diabetes will increase to 7.7% by 2030 [2]. Diabetes, a growing disease not only at the global level, but more specifically in developing counties and in the Texas Rio Grande Valley (RGV) has been of great concern [2,3]. Patients with diabetes have shown upregulation of oxidative stress, cytokines, and proteins that are significantly altered by the hyperglycemic conditions, specifically the eye [1]. Diabetic diseases that occur in the eye include diabetic retinopathy (DR), diabetic macula edema (DME), cataracts, glaucoma [4]. In the present study, we will focus on the molecular pathway associated with etiology of DR.

DR has a plethora of pathways associated with hyperglycemia-induced retinal damage that including increased oxidative stress [5], advanced glycation end products (AGEs) accumulation [6], change in the level of transforming growth factor beta (TGFβ) [5], increased inflammation responses [7], and vascular endothelial growth factor (VEGF) [8]. VEGF associated with proliferative diabetic retinopathy (PDR) is the hallmark of this disease [9]. High glucose induced increased levels of VEGF have been associated with increased inflammatory cytokine found in DR in multiple cell lines. VEGF has been shown to increase angiogenesis and proliferation of blood vessels. Our previous studies in cone photoreceptors have shown a significant increase in oxidative stress and increased levels of VEGF secretion [8]. Rod and Cone photoreceptors have elevated metabolism; thus, highly sensitive to oxidative stress from hypoxia. Low oxygen level, however, may lead to oxidative damage to cone cells leading to cell death and increasingly alarming levels of other cytokines and proteins. Different retinal pathways are currently under investigation to determine how we can target specific markers to inhibit these exacerbated effects that cause vision loss in the retinal diseases.

In oxidative stress conditions, the protein structures are changed, playing a significant role in the pathogenesis of DR. Previous studies showed that low levels of oxidative stress induced inflammation and apoptotic of retinal cells. In contrast, high levels of oxidative stress may lead to detrimental effects such as DNA mutations. Moreover, studies have shown that hyperglycemia can cause increasing oxidative stress in animal models; significantly increased levels of VEGF secretion [5]. Oxidative stress associated with DR, has potential to cause inflammation, cell death, apoptosis, and damage to microvasculature in the retina [5]. Kurihara et al. suggested that though photoreceptors may not be directly impacted by the oxidative stress (hypoxia) they can induce the promotion of degeneration of the photoreceptors as seen in retinas of mice exposed to hypoxic conditions [6]. Additionally, it has been confirmed by Wellard et al., that rat retinal photoreceptors undergo apoptosis, as a result of hypoxic exposure; Wellard et al. also included that mouse models also showed similar results [7].

Photoreceptors are responsible for secreting interphotoreceptor retinol binging protein (IRBP), an antioxidant to maintain balance of ROS. However, in hyperglycemic conditions, ROS in increased, leading to IRBP synthesis inhibition, ultimately leading to photoreceptor cell death. Overall, the retina is an important generator of ROS and is known to be in resulting retinal diseases [8]. This gives us insight on how oxidative stress plays a significant role in the pathogenesis of DR. In low ROS conditions there are alteration of protein structures leading to changes protein function some of which associated with signal transduction pathways related to proliferation, inflammation, apoptotic and gene expression. Animal studies have previously shown that an increase of ROS generation is accumulated in the presence of hyperglycemia conditions [7]. It is noted that the association of increased ROS is linked to mitochondrial DNA damage. Retinal endothelial blood barrier damage caused by ROS leads to the photoreceptor damage. Being that oxidative stress is increased due to underlying diabetic conditions, understanding the molecular pathway of oxidative stress on 661W cone photoreceptors is needed [8,10–12].

TGFβ superfamily is responsible for many of the cellular processes including proliferation, differentiation, cell death, adhesion, and migration. The TGFβ superfamily in mouse models play an important role in the development as well as mediating anti-proliferative effects in epithelial cells, regulation of immune, and healing responses. Many diseases have been associated with TGFβ family signaling pathway. SMADS, involved in the signaling cascade of TGFβ pathway, directly send signals from cell surface transmembrane receptors to the nucleus through intracellular proteins. Inhibition of SMADs, induced by TGFβ signaling resembling a negative regulator of the pathway by binding to the type 1 receptor, thus competing with R-SMADS and blocking the phosphorylation from occurring [13]. TGF alpha and TGFβ are both components involved in the pathway leading to diabetic retinopathy [14]. Lee et al. studied the effects TGFβ and the notch signaling pathways, in conjunction with SMAD3 pathway in the proliferation of zebrafish. TGFβ is said to enhances extracellular matrix production after injury; proliferation of is crucial for the regeneration of these cells.

Researchers have tested TGFβ1 and TGFβ3 in various zebrafish models. Zebrafish were previously tested and had significant release of different growth factors and cytokines after injury, possibly caused by stress, and exacerbation of effects that would ultimately lead to cell death. Zebrafish have a high density of cone photoreceptors [15]. Previously studies, have shown that tissue infarction and impaired heat regeneration is possible by chemically inhibiting the TGFβ pathway using a chemical inhibitor SB431542. This inhibitor blocks the TGFβ type 1 receptor of TGFβ. Additionally, results indicate Smad2/3 mediated TGFβ signaling acts to inhibit proliferation of neuronal progenitors following photoreceptor destruction in zebrafish retinas. TGFβ signaling pathway, however, remains unclear. By inhibiting SB431542 during retinal regeneration after inducing MNU induced photoreceptor degeneration, they also observed retinal regeneration after the inhibition of the TGFβpathway [16]. TGFβ was observed to determine how TGFβ affected the proliferation and regeneration of neuronal retinal glial cells in the retina of zebrafish by Cia in 2020. They associated TGFβsignaling pathway with downstream SMAD pathways, leading to proliferation. When inhibiting TGFβreceptor, inhibition of the pathway TGFβ as well as inhibition of its effects caused by SMAD; additionally, it was shown that TGFβ could stimulate the expression of SMAD. Ultimately, if inhibition of TGFβ1 or 3 will mitigate cell proliferation. All these factors can lead to the damage and destruction of vessels in the retina, photoreceptors ultimately leading to enhanced retinal neovascularization as seen in DR [16,17].

## Materials and Methods

### Cell culture

Cone photoreceptor (661W) cells were obtained from Baylor College of Medicine, Houston. They were maintained at 37° Celsius in 5% carbon dioxide and 95% oxygen in culture. Dulbecco’s modified eagle medium (DMEM) [Corning catalog # 26520011], culture media consisted of 10% fetal bovine serum [Corning catalog # 35–010-CV] and 1% anti-anti [Gibco catalog # 15240–062]. During the culturing processes PBS (1x) was used for cleaning and 0.25% trypsin-EDTA [Gibco catalog # 25200–072] was used for the detachment of cells. Cells were passages upon confluency and cultured in 6 wells with 300,000 cells per well for 24 hours before pretreatment or treatment.

### Treatments and pathway inhibitors

Glucose was prepared using 5.5mM normal glucose (NG) and 30mM high glucose (HG). Cobalt (II) Chloride (CoCl_2_) 300uM was used to induced hypoxia-like conditions. Cells were pretreated 24 hours before treatment with either SMAD 3 inhibitor^1^, SIS3 [Millipore Corp. catalog # 3290282] or TGFβ R1 Kinase Inhibitor^2^ [SB431542, Millipore Corp. catalog # 3498270] dissolved in DMSO.

### Viability of 661W cells

Cell counts were carried out using a Hemocytometer with Trypan blue staining to determine the number of viable cells after treatment.

### Protein quantification

To determine the VEGF concentration in the conditioned media, mouse VEGF ELISA kit was used [R&D systems, catalog number: MV000]. After 24-hour treatment, 1mL of the condition media collected from the plates for each treatment was stored at −80°C or quickly analyzed through VEGF ELISA Enzyme-linked immunosorbent assay. The data produced by the ELISA consists of a standard curve and concentrations of VEGF per sample (pg./mL).

### Statistical analysis

Mean of three replicates were calculated. An ANOVA to determine the differences between one or more group means from each trial was done. One-way ANOVA was used to determine significant effect of independent variable (treatment) on dependent variable (VEGF secretion/cell viability).

## Results

### Effects of hypoxic on 661W cone photoreceptor cells

**Figure 1:**
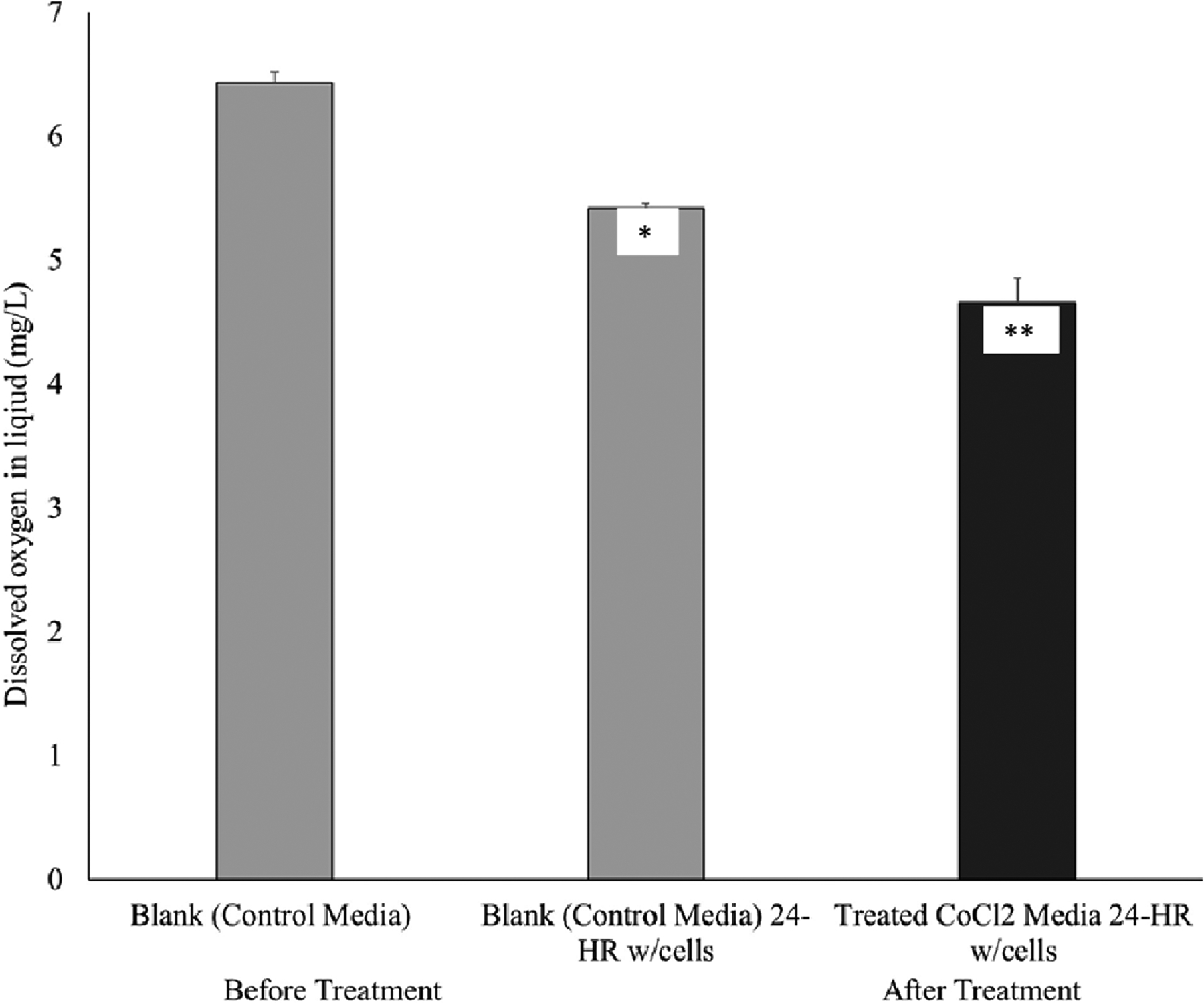
Dissolved oxygen (DO) levels (mg/L), in conditioned DMEM, were reduced with use of Cobalt (II) Chloride (CoCl_2_). Levels of DO were measured in DMEM before treatment and after treatment with cells with and without the induction of hypoxia-induced conditioned media with use of 300uM Cocl2 in 661W cone photoreceptor cells, following 24-hour. Error bars represent the standard error of triplicate samples (*p<0.05, **p<0.001).

**Figure 2:**
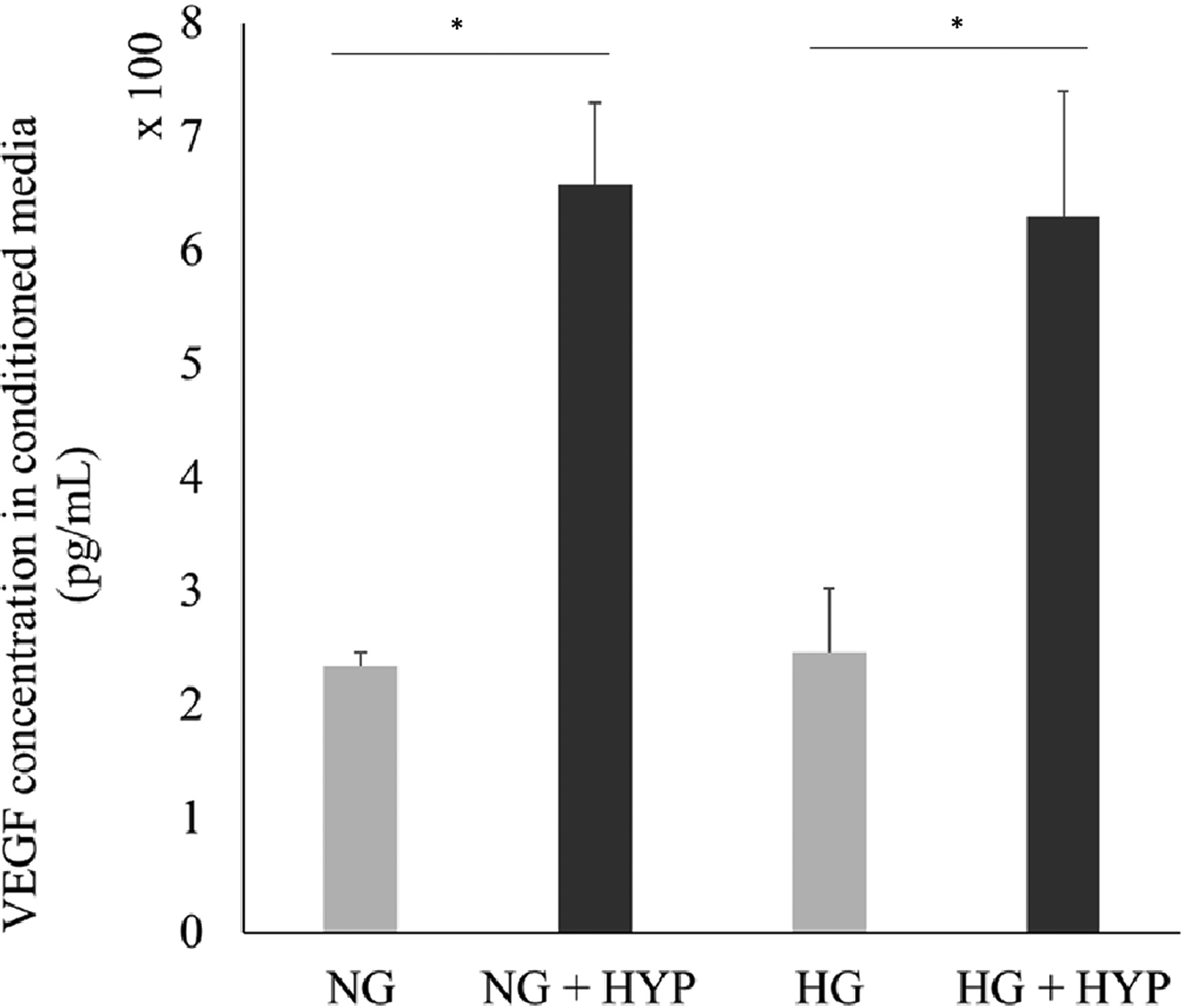
Hypoxic induced elevation of VEGF secretion in 661W cone photoreceptor cells. Cells were exposed to 300uM CoCl_2_, hypoxic conditions (HYP) for 24-hours, then conditioned media was collected and analyze to determine amount of VEGF secreted by cells in both normal (NG) and high glucose (HG). Error bars represent the standard error of triplicate samples (*p<0.05).

**Figure 3:**
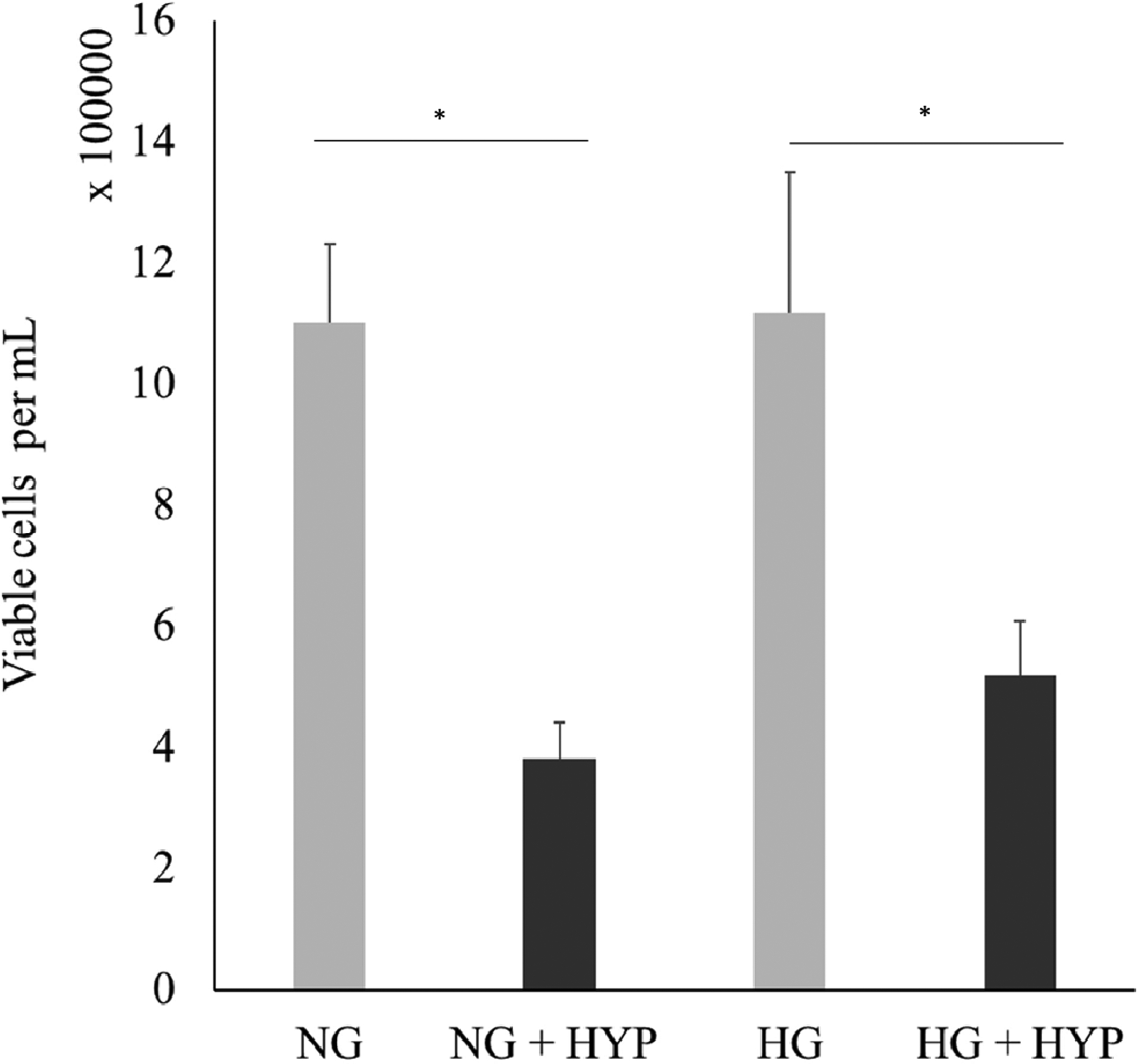
Hypoxia induced reduction of 661W cone photoreceptor cells. Cells were treated with 300 uM Cobalt (II) Chloride (CoCl_2_) for 24 hours to induce hypoxia, in both normal (NG) and high glucose (HG). Following treatment, viable cell number was determined with the use of a hemocytometer cell counter after treatment with trypan blue. Error bars represent the standard error of triplicate samples (*p<0.05).

To stimulate the hypoxic environment for cellular process, 300uM cobalt (II) chloride (Cocl_2_) was used to cells that were previous grown to confluency. As shown in Figure 1, treatment of CoCl_2_ resulted in a significant decrease in dissolved oxygen in conditioned media. Figure 2 shows VEGF secretion significantly increased from hypoxia treatment in both low and high glucose conditions. Additionally, Figure 3 shows that viable cells exposed to hypoxic conditions resulted in a significant decrease in cell number (in both high and low glucose treatment groups) after a 24-hours treatment period.

### Involvement of TGFβ pathway in 661W cone photoreceptor cells in oxidative stress

**Figure 4:**
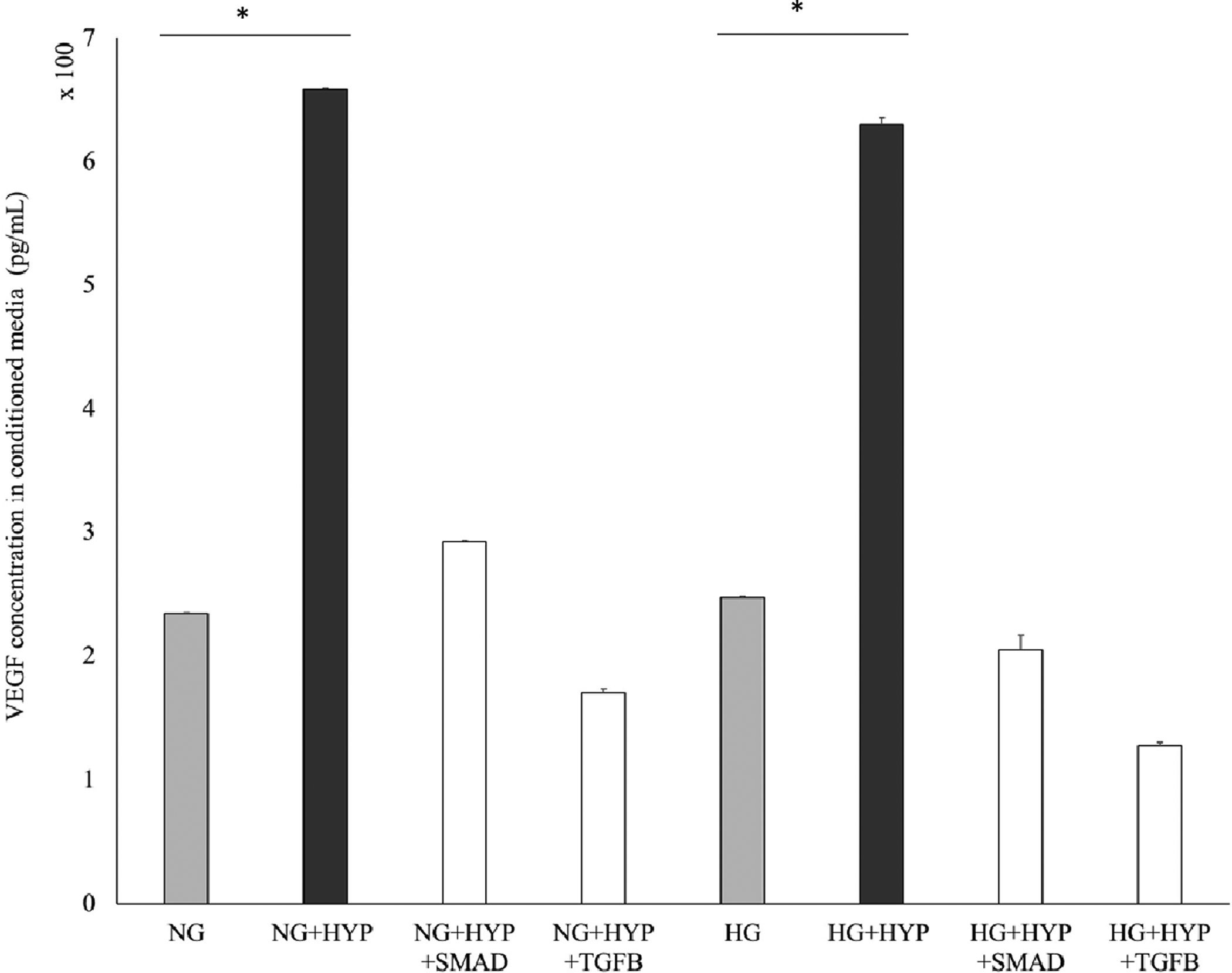
TGFβ signaling pathway involvement in hypoxia-induced VEGF secretion. Cells were pre-exposed to either 10uM SMAD 3 inhibitor, SIS3 (SMAD) or TGFβ R1 kinase inhibitor, SB431542 (TGFβ) for 24 hours before treatment. Cells were then treated with glucose concentration including 5.5mM normal glucose (NG) or 30mM high glucose (HG) and with or without Cobalt (II) Chloride (CoCl_2_) for 24 hours. Conditioned media samples were collected and analyze through ELISA assay to determine VEGF secretion in 661W cells after treatment. Error bars represent the standard error of triplicate samples (*p<0.05).

**Figure 5:**
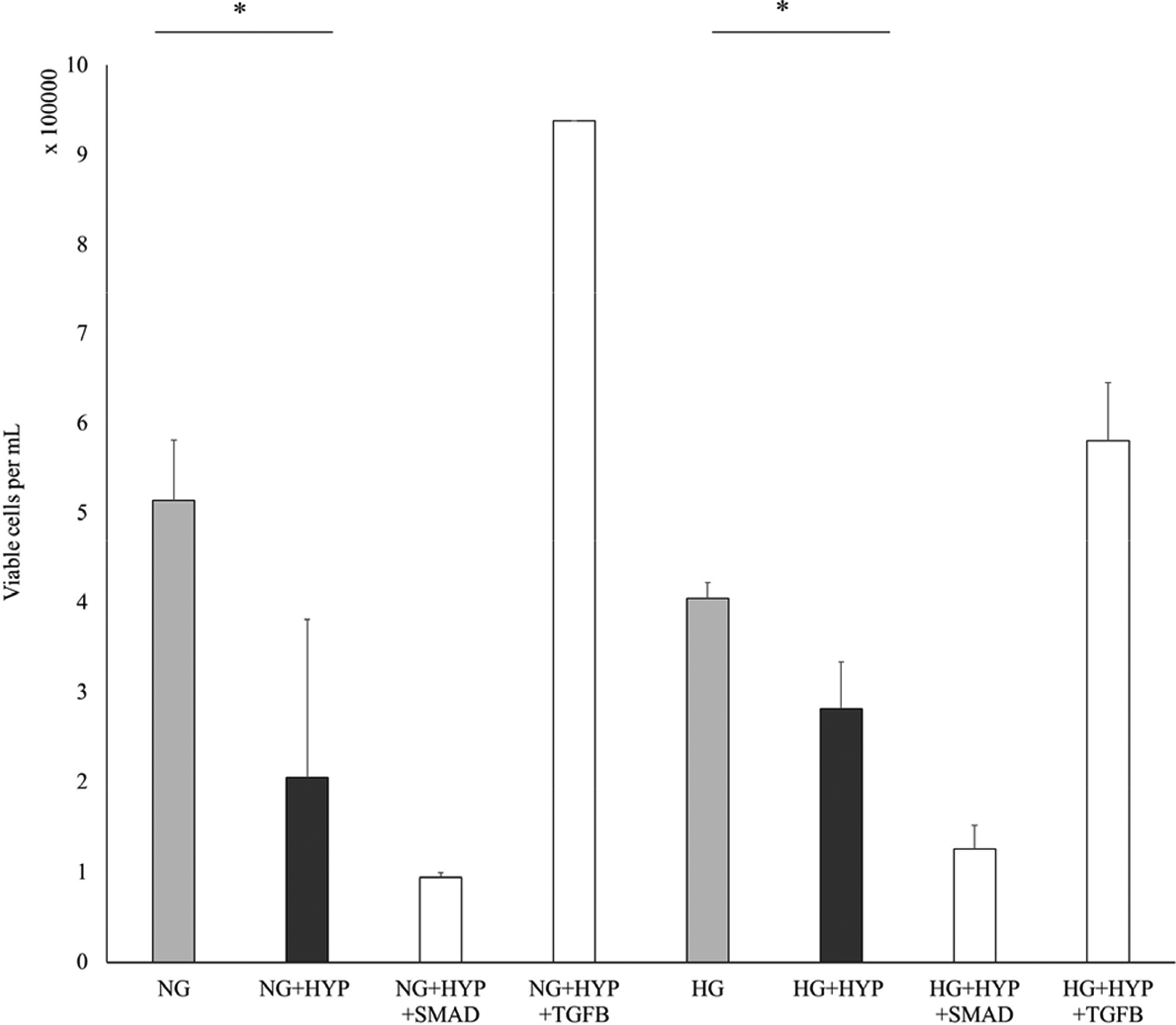
TGFβ signaling pathway involvement in hypoxia-induced reduction of cell number. Cells were pre-exposed to either 10uM SMAD 3 inhibitor, SIS3 (SMAD) or TGFβ R1 kinase inhibitor, SB431542 (TGFβ) for 24 hours before treatment. Cells were then treated with glucose concentration including 5.5mM normal glucose (NG) or 30mM high glucose (HG) and with or without Cobalt (II) Chloride (CoCl_2_) for 24 hours. Following treatment, viable cell number was determined with use of a hemocytometer after treatment with trypan blue. Error bars represent the standard error of triplicate samples taken for statistical analysis (*p<0.05).

Data shown in Figure 4 indicate inhibitor 1 (SMAD 3/SIS) and inhibitor 2 (TGFβ receptor 1 kinase inhibitor) both returned the VEGF concentration to the level prior to hypoxia treatment (in both low and high glucose). However, only inhibitor 2 return cell number to the level similar or higher to cell numbers prior to hypoxia treatment (Figure 5). In contrast, inhibitor 1 did not restore cell numbers to the level prior to hypoxia treatment following 24-hour treatment.

## Discussion

Previous studies suggested that in the presence of hyperglycemia growth factors increase, significantly inducing transduction pathways that led to AGEs and oxidative stress which leads to retinal hypoxia and an increase in growth factors specifically, VEGF [15]. We have previously investigated the secretion and expression of VEGF in human retinal pigment epithelial (HRPE) cells and their involvement with the TGFβ signaling pathway. Hyperglycemic conditions induced increased levels of VEGF 121, 165, and 189 due to an upregulation of the TGFβ pathway [18]. Human retinal pericytes (HRP) have also been used to test the effects of TGFβ1 and TGFβ2 in the induction of BIGH3 protein and apoptosis, suggesting that the increase of the BIGH3 causes loss of retinal pericytes during early events in diabetic retinopathy [19]. These important finding support the ideology that cells can potentially have a degree of inflammatory response to significantly change the amounts of VEGF secretion secreted via photoreceptors. Moreover, these studies, suggested that TGFβ pathway had significant impact on the VEGF secretion by HRPE cells. In this study, we investigated the involvement of TGFβ signaling pathway after cells were exposed to both glucose and hypoxic conditions to determine if the TGFβ pathway was responsible for the increase in VEGF secretion. Although there was no distinct effect of high glucose induction of VEGF secretion and viable cell number, difference hypoxic conditions induced a significantly elevated VEGF level and a significant reduction in cell number. With the inhibition of TGFβ, we further observe that there a full recovery (from hypoxia treatment) of cell number (using the TGFβ R1 kinase inhibitor) and VEGF level. In contrast, SMAD 3, SIS inhibitor of the same pathway further decreased VEGF level and restored cell number to a level much higher than those resulted from hypoxia treatment, suggesting involvement of addition mechanism/pathways. Further investigation will be needed to identify these novel pathways.

## Conclusion

Cone photoreceptors (661W) exposed to high glucose for 24 hours, did not induced changes in VEGF secretion and viable cell number cells. However, hypoxia induced a significant increase in VEGF secretion and decreased the number of viable retinal cells. These effects were reversed by the addition of TGFβ pathway inhibitors, suggesting the involvement of this signal transduction pathway. Further translational research studies will provide evidence to identify appropriate and effective pharmaceutical targets in this molecular pathway to mitigate the development of DR.

## Abbreviations:

DR: Diabetic Retinopathy
VEGF: Vascular Endothelial Growth Factor
TGFβ: Transforming Growth Factor Beta
PDR: Proliferative Diabetic Retinopathy
CoCl2: Cobalt (II) Chloride
